# Accuracy of Conventional and Digital Methods of Obtaining Full-Arch Dental Impression (In Vitro Study)

**DOI:** 10.7759/cureus.29055

**Published:** 2022-09-11

**Authors:** Hassan A Husein, Mhd. Luai Morad, Shaza Kanout

**Affiliations:** 1 Department of Fixed Prosthodontics, Faculty of Dental Medicine, Damascus University, Damascus, SYR

**Keywords:** extra-oral scanner, 3d model, digital impression, conventional impression, intra-oral scanner

## Abstract

Introduction

The current gold standard is a conventional impression made with various impression materials and trays and results in a gypsum cast. With the development of milling and printing materials in dentistry, especially zirconia, a digital model has become increasingly important.

Objectives

To compare the accuracy of the conventional impression scan (CIS), gypsum cast scan (GCS), and digital impression scan (DIS) to obtain a full-arch digital model.

Materials and methods

A resin reference cast was fabricated. It was scanned by an extra-oral scanner to measure its accuracy as a reference scanner. Eight conventional impressions of the reference cast were taken by polyvinyl siloxane and scanned. After that, they were poured with type IV dental stones and scanned too. The reference cast was scanned by an intraoral scanner eight separate times. Digital models within each group were superimposed individually to measure precision. In addition, each model from each group was superimposed on one model from the reference scanner precision group to measure trueness.

Results

The reference scanner showed the highest accuracy among groups with a precision of 1.5±0.8 µm and a trueness of 5.5±1.9 µm (P<0.006), while precision values of gypsum cast were 8.1±1.7 µm and trueness values were 9.3±2.6 µm (P<0.012). Conventional impressions showed a precision of 14.06±2.01 µm and a trueness of 16.15±2.07 µm (P<0.012). Digital impressions were the least accurate among the groups, as precision values were 38.22±15.23 μm and trueness values were 35.19±8.7 μm (P<0.006).

Conclusion

The gypsum cast scans showed the highest accuracy, followed by the conventional impression scans, and finally the digital impression scans, with no clinical significance.

## Introduction

Manufacturing prostheses using indirect methods successfully can be an accomplishment in dental practice, especially inlays, crowns, and bridges. Using these indirect methods, prostheses can be accomplished with most of their lab procedures away from the dental clinic [1]. The impression has its importance in various aspects of dentistry; to perform various procedures like diagnosis, model analysis, and fixed prosthesis fabrication. The use of gypsum models is not only essential but routine practice in prosthodontics [2]. The conventional impression, which is made with elastomeric impression materials, is considered a gold standard, taking it using various techniques that show high accuracy results in the literature [3-5].

Different materials and impression techniques were used to accomplish high-accuracy impressions. Deviations were evaluated in vitro by calculating line distances between the reference model and the gypsum cast. The impression is verified clinically by fitting the final restoration [6-9].

The importance of digital models has increased after the evolution of CAD/CAM systems [10], especially after developing intraoral scanners [10-12], which scan the oral structures and produce a virtual model. Virtual models are a reliable way to archive study models, producing durable images without any fear of loss or damage to the original models [13].

Evaluating the accuracy of the digital impressions by calculating line distances is not preferable. However, verifying the accuracy can be done by superimposing digital models with a reference digital model, then calculating three-dimensional distances to show deviations between the impression and the reference cast for each point on the cast's surfaces [14].

Nicholls defined distortion as the relative or absolute motion of a point or group of points with respect to one or more reference points. The distortion is divided according to the reference point into: absolute distortion when the reference point is external, and relative distortion when the reference point is located inside the system. Resorting to relative distortion analysis yields data that are closer to clinical than absolute distortion analysis [15].

Precision and trueness are two terms used to define accuracy [16]. Precision describes the symmetry of repeated measurements with respect to each other. As the precision is higher, the measurement is more predictable [14,17]. On the other hand, trueness describes how the measurements are close to the real dimensions of the measured structures [4,14].

The accuracy of the impressions is affected by many factors, such as occlusion type and tooth placement [18,19]. As well as obtaining accurate 3D-printed models is related to the accuracy of the digital models [20], which are also useful in facilitating communication between dentists to evaluate and study cases and predict results through simulation [13]. There are many ways to get a digital model by using extraoral and intraoral scanners.

The aim of this study was to assess the accuracy of conventional impression scans (CIS), gypsum cast scans (GCS), and digital impression scans (DIS). The null hypothesis states that there is no difference in accuracy between CIS, GCS, and DIS.

## Materials and methods

**Figure 1:**
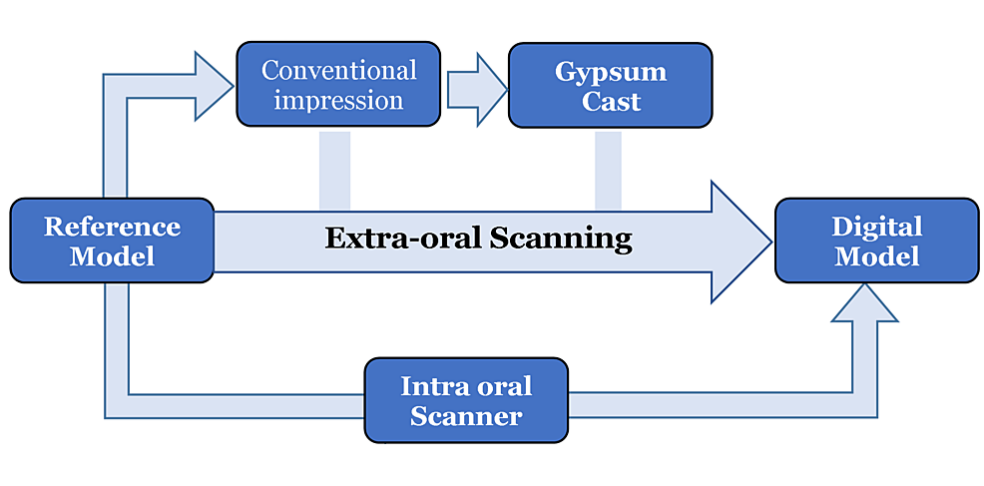
Methodology of study

A resin reference cast of an upper jaw was designed by Exocad software (Fraunhofer, Munich, Germany) and printed by a Zyrlux 3D printer (DENTIS, La Palma, CA). It was scanned by the Identica T300 (Medit, Seoul, Korea) extraoral reference scanner eight separate times in the same position to assess precision (RCS-prec). The reference cast was re-scanned eight more times, by rotating the cast 45° in a horizontal plane around the z-axis with minor angles of 10° to 20° around the x- and y-axis each time before scanning, to assess trueness (RCS-true). Then eight conventional impressions were taken by PVS (3M, Germany), and the impressions were disinfected for 10 min and stored for eight hours prior to scanning by the reference scanner (CIS). After that, it was poured with type IV dental stone and scanned by the same scanner (GCS). Eight digital scans were taken by setting the tip of the i500 (Medit) intra-oral scanner above the occlusal surface of the right third molar and pressing the button to start the scanning process successively to the right canine, then the tip of the device was tilted towards buccal and lingual surfaces in successive movements up to the left canine, back to the occlusal surface until the left third molar, then we moved to scan the lingual and buccal surfaces of the teeth, before pressing the stop button (DIS) (Figure 1). Finally, digital model data for all groups were exported as standard triangulation language files (STL).

**Figure 2:**
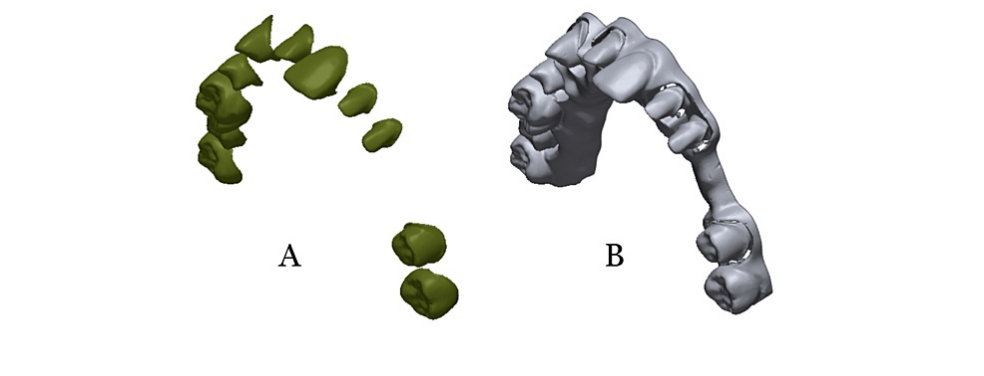
(A) Measured DATA and (B) reference DATA

To evaluate the accuracy, Geomagic® Control X™ had been installed to read STL files. A cut was made at a level of 2 mm below the cervical lines of the teeth in the model, which was included as reference data (Figure 2A). A cut at the level of the cervical lines for unprepared teeth and at the level of the finishing lines for prepared teeth was included as measured data (Figure 2B). To assess precision, superimpositions were used for each of the two digital models within the same group. Therefore, each group contains 28 superimpositions (n=28). Superimpositions were made by entering the data of the first model as reference data and the second model as measured data and making an initial superimposition by pressing the Initial Alignment button. After that, press the Best Fit Alignment button. The shortest distance between each point on the surface of the first model and a corresponding point on the surface of the second one was calculated. This distance could be negative or positive. The 3D compare button was pressed to get the different color maps of the superimposition. After the range [+20, −20] μm was selected as the optimal values, and the range [+120, −120] μm as the highest and lowest values, a report was generated from the Generate Report button, which contains the differences in color images of the superimposed models for visual analysis. Finally, the report was exported as a pdf file. Deviations of superimposed models (gap distances) of the complete dental arch were exported as csv files. Then inserted them into an excel sheet to determine the highest and lowest 5% of the deviations' values for each superimposition and delete them. An average of the absolute values of the remaining 90% for each superimposition was calculated to represent the average of the deviations for each superimposition.

**Figure 3:**
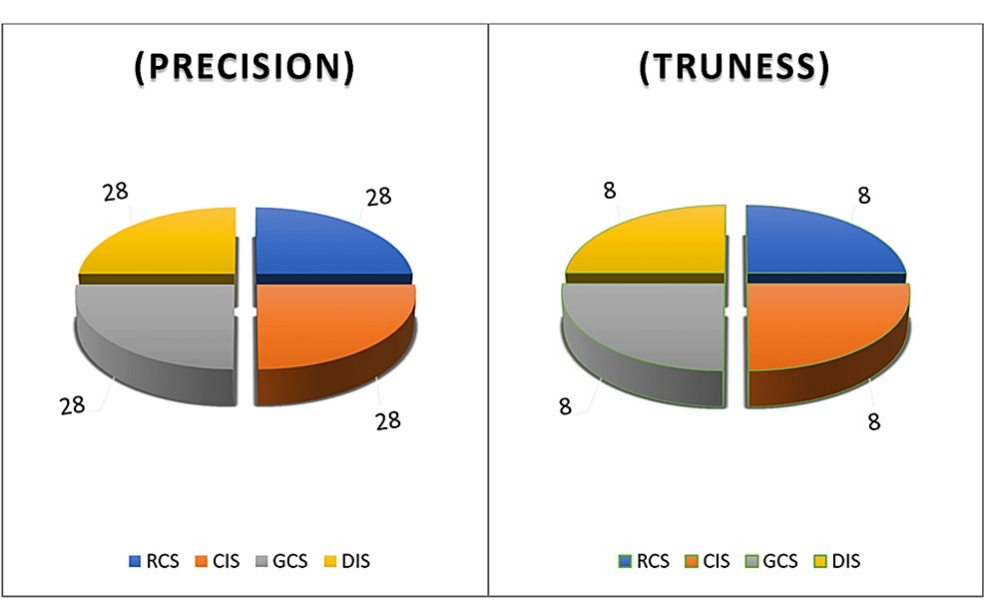
Sample distribution

To assess trueness, the previous process was repeated where one digital model file from each RCS-prec group (in the same position) was used as reference data in all superimpositions for all groups, so that each group contains eight superimpositions (n=8) (Figure 3).

Statistical analysis

The Shapiro-Wilk and Levene tests were used to assess the normal distribution and homogeneity of the sample. For precision assessment, the Kruskal-Wallis test was used. After that, pairwise comparisons with Bonferroni correction were used to find the differences between groups. The Welch test was used to assess trueness for all study groups. To analyze the differences between groups, the Games-Howell test was used. All statistical tests were performed using SPSS26 software (IBM Corp, USA) at a significance level α=0.05.

## Results

The trueness and precision measurements among all groups are shown in Table 1.

**Table 1:** Descriptive statistics All table values in µm

	Groups	Mean	SD	Median	Max.	Min.
Precision (n=28) for each group	MCS	1.50	0.86	1.66	0	3.42
	CIS	14.05	2.01	13.88	11.01	17.45
	GCS	8.17	1.76	7.52	6.25	13.36
	DIS	38.22	15.23	34.78	18.13	74.28
Trueness (n=8) for each group	MCS	5.55	1.98	5.85	1.66	8.06
	CIS	16.15	2.07	15.58	13.43	19.40
	GCS	10.22	1.06	10.52	8.18	11.51
	DIS	35.19	8.70	33.40	24.28	47.52

Accuracy of the reference scanner

**Figure 4:**
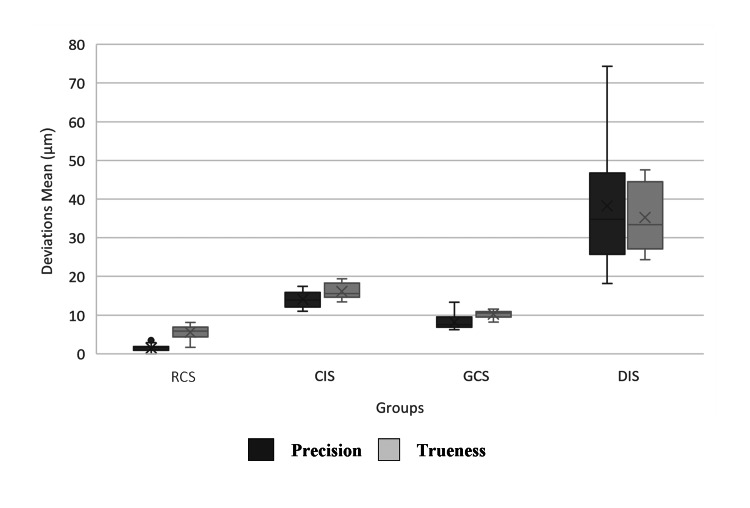
Boxplot of accuracy

**Figure 5:**
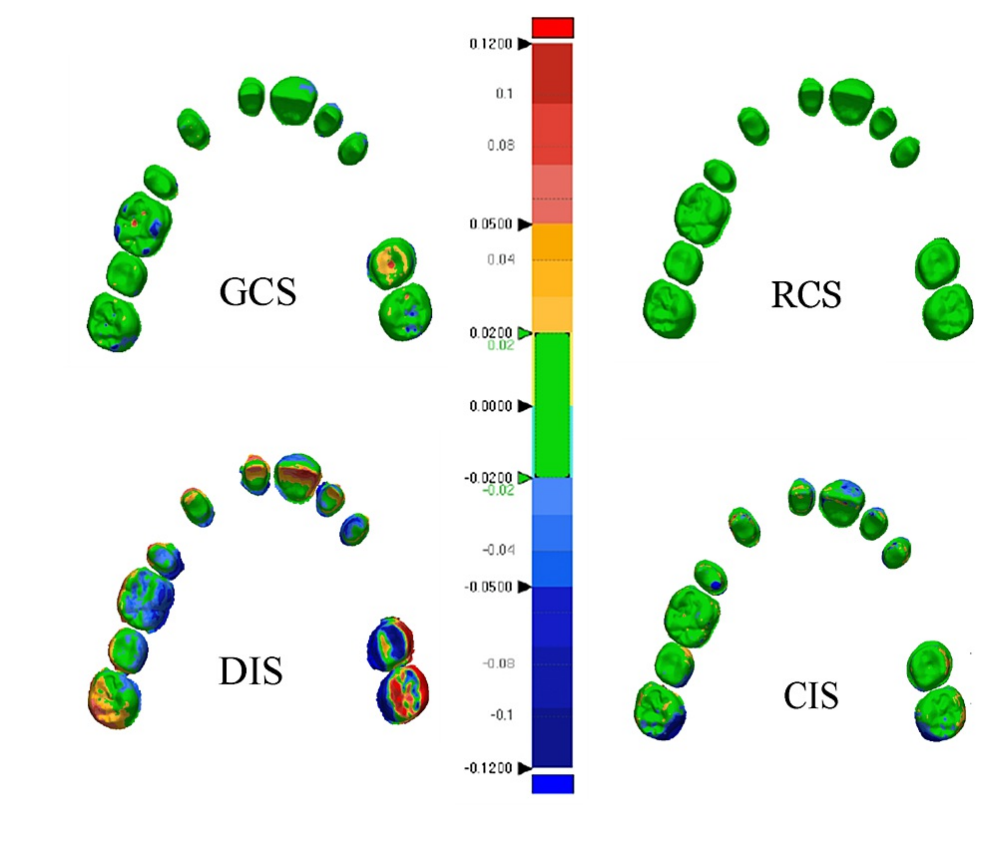
Color images of precision

The RCS-prec group had statistical significance compared to the other groups (P<0.006) (Figure 4). The superimposition color map analysis showed that the deviations ratios mean of less than 10 µm was 98.9%, and the deviations ratios of less than 20 µm ranged from 99.69% to 100%, as the highest difference was noticed at the incisal edge of the central incisor (21 µm) (Figure 5).

**Figure 6:**
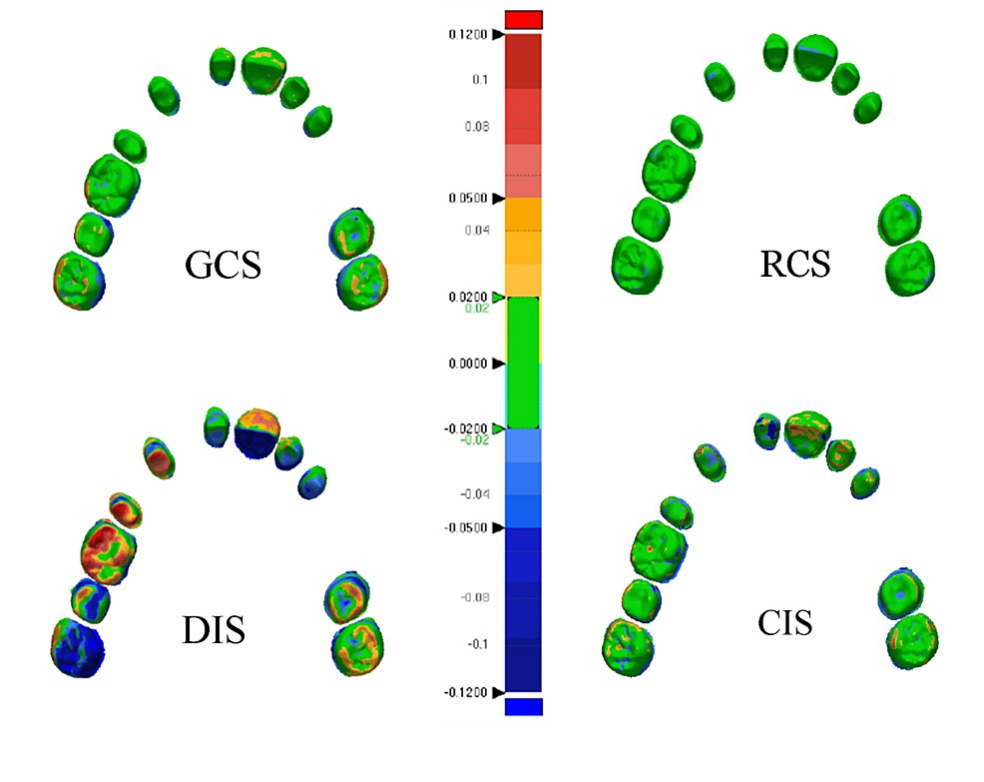
Color images of trueness

The RCS-true group had statistical significance compared with the studied groups (P<0.001) (Figure 4). The superimposition color map analysis showed that the deviations ratios mean of less than 10 µm was 77.19%, and the deviations ratios of less than 20 µm ranged from 87.96% to 100%, as the deviations ratios mean of greater than +20 µm was 5.62%, which occurred in the deep central grooves and palatal surfaces of the anterior teeth (14 µm). In addition, the deviations ratios mean of greater than −20 µm was 0.32%, occurring in the head of the cusps and in the cutting edges closer to the outer slopes (−33 µm) (Figure 6).

As a result, the reference scanner is capable of evaluating the accuracy of conventional and digital impressions.

Accuracy of conventional impressions

CIS-prec had a statistically significant difference compared to other study groups (P<0.002). The superimposition color map analysis showed that deviations of less than 20 μm ranged from 57.80% to 75.88% with a mean of 66.91%, as the differences outside the field [−20, +20] µm were not concentrated in specific areas. However, it was noted that these deviations were in the distal areas of the arch, especially the distal and palatal surfaces of the last molar and the buccal surfaces of the anterior teeth, in more than half of the sample. The deviation was equal to 90 μm at the palate surface of the second molar (Figure 5).

CIS-true had a statistically significant difference compared to other study groups (P<0.002). Moreover, the superimposition color map analysis showed that deviations of less than 20 μm ranged from 53.81% to 68.11% with a mean of 61.33%. The deviations ratios mean of greater than +20 µm was 18.69%, which occurred on the palate surfaces of the anterior teeth and the buccal surfaces of the posterior teeth. Maximum differences of up to 162 μm occurred on the palatal surface of the central incisor. The deviations ratios mean of greater than −20 µm was 19.95%, not occurring in specific areas. The difference value of −72 μm occurred on the palatal surface of the second molar (Figure 6).

Accuracy of gypsum casts scans

GCS-prec showed a statistically significant difference compared to other study groups (P<0.012). For the superimposition color map analysis, deviations of less than 20 µm ranged from 68.28% to 93.77% with a mean of 87.53%, as the deviations ratios mean of out-of-field [−20, +20 μm] was 12.57% and did not occur in specific areas. However, deviations were observed in the central grooves of unprepared posterior teeth with a different value of (96 μm) in the central fossa of the second right molar (Figure 5).

For trueness assessment, GCS-true had a statistically significant difference compared to other study groups (P<0.001). By the superimposition color map analysis, it was found that deviations of less than 20 μm ranged from 69.05% to 88.88% with a mean of 79.13%. The deviations ratios mean of greater than +20 μm was 10.53%, mostly occurring in the buccal surface of the central incisor, buccal and distal areas of the molars, and the left side more than the right one. That is, deviations have increased by moving away from the center of the model. The highest deviation, of 51 μm, was located at the distal surface of the second molar. The deviations ratios mean of greater than −20 µm was 10.31%, occurred in the palatal surfaces of the same areas. The highest deviation of −45 μm was located at the palatal surface of the second molar (Figure 6).

Accuracy of digital impression scans

DIS-prec showed a statistically significant difference compared to other study groups (P<0.006). The superimposition color map analysis showed that deviations of less than 20 μm ranged from 16.59% to 54.44% with a mean of 34.25% as the deviations ratios mean of out-of-field [−20, +20 μm] was 65.73% and was generalized on the entire dental arch. Deviations exceed 180 µm in more than one area (Figure 5).

DIS-true had a statistically significant difference compared to other study groups (P<0.006). In the superimposition color map analysis, deviations of less than 20 μm ranged from 23.89% to 41.33% with a mean of 32.35%. The deviations ratios mean of greater than +20 μm was 35.31. These deviations occurred on the buccal surfaces of most of the teeth and the buccal and distal surfaces of the last molar in more than two-thirds of the sample and exceeded 183 µm. The deviations ratios mean of greater than −20 μm was 32.31%. The highest deviations were located at the palate surfaces of the teeth in more than two-thirds of the sample. The visual analysis of trueness showed low deviations in the anterior teeth, with the highest difference in premolars and molars compared to the reference model. Irregular deviations were noticed in the distal areas of the end of the dental arch equal to −205 μm (Figure 6).

## Discussion

The reference scanner showed the highest accuracy with a precision of 1.50±0.86 μm, and a trueness of 5.55±1.98 μm, followed by the GCS group with a precision of 8.17±1.76 μm, and a trueness of 9.34±2.62 μm, and the CIS group with a precision 14.05±2.01 μm and a trueness 16.15±2.07 μm. Finally, the DIS group with a precision of 38.22±15.23 μm and trueness of 35.19±8.70 μm.

To assess the trueness of the reference scanner, the reference cast position has changed at a 45-degree angle around the z-axis in each scan, changing the coordinates of all model surface points in the x-y-z axis, revealing mathematical errors and calibration errors in the scanner by comparing the resulting digital model with the initial position. However, these errors can be avoided by scanning all study groups in a standardized pre-calibration position. Repeating scans in the same initial position gives us the precision value.

Precision measuring tools are called digital converters, digitizers, or scanners, and include a tactile probe, profile projector, imaging measuring machines, video lasers, or computer coordinates that can be used for Cartesian coordinates [21]. The researchers presented the idea of adding pre-calibration objects (balls-metal beams) to the reference cast and comparing its dimensions after scanning them with digital scanners [22,23]. A new method of evaluating accuracy has been introduced, using a high-resolution reference digital scanner and a software program that superimposes scans and compares them to treat them statistically [14]. Other studies used geometric forms to verify coordinate measuring machines (CMMs) and showed high trueness and precision for these devices. However, these CMMs acquire only a small number of points from the model surface. Additionally, for a precise model with CMM, knowledge of the surface shape before scanning is necessary. Therefore, the extra-oral scanning technique was adopted by a precise scanner based on the Phase Shifting Triangulation technique, as its accuracy was evaluated by assessing the trueness and precision of this scanner before evaluating other groups. This was done using the STL files reading program and identifying differences to collect them in a CSV file, then arrange them in an excel sheet. Finally, appropriate statistical analysis was done, with visual analysis of color differences in image resulting from superimposing scans. The intra-oral scanner that uses video-type scanning based on triangulation technology was used, which is able to capture moving objects and, therefore, scanning can be continued while the object is in motion. Triangulation uses a 3-camera pattern in order to capture 3D images [24].

A cut in the reference data for the superimposition was made 2 mm below the level of the teeth's cervical line to reduce superimposition errors when using the best-fit alignment. In the measured data, a cut was made at the level of the cervical line of the teeth to determine the deviations of the teeth areas only.

The highest and lowest 5% of these differences were not included for comparison, due to scattered points on the surface that may contain errors, as there may be areas of low density and areas with a high density greater than 80 degrees that cannot be accurately scanned by the reference scanner, where they cannot be relied upon to make comparisons. This method is more accurate than using the root mean square (RMS), which represents only 66% of the measured differences, which was mainly used in previous studies [14].

The extra-oral scanner was adopted as the standard reference scanner as it had the highest accuracy among the study groups with a statistically significant difference and showed the lowest value for the differences mean for RCS-prec of 1.5±0.86 µm (P<0.006), and RCS-true of 5.55±1.98 µm (P < 0.001), which is consistent with a study by Ender et al., as it was 1.6±0.6µm for precision and 5.3±1.1µm for trueness [14].

The field [+20, −20] μm was selected as the ideal field, according to what is mentioned in Craig's Restorative Dental Materials book "The impression should be compatible with gypsum products so the 0.02 mm line is transferred to gypsum die materials" [25].

The deviations ratios mean of less than 20 μm in RCS-prec was 99.91%, with the highest difference located in the incisal edge. The deviations ratios mean of less than 20 μm in RCS-true was 94.79%, the highest positive deviation occurred in the deep central grooves and palatal surfaces of the anterior teeth, and the highest negative value was in the head of the cusps and incisal edges closer to the outer slopes. This slight deviation may be due to the sharp slope between the buccal and palatal surfaces at the incisal edges of the anterior teeth and between the internal slopes of the occlusal surfaces of the posterior teeth.

The null hypothesis, which states that there is no difference in accuracy between study groups, was rejected.

The GCS group showed the highest accuracy, followed by the CIS group, and the DIS group, with a statistically significant difference between all groups. The differences mean in GCS-prec was 8.17 ± 1.76 µm (P<0.012). The deviations ratios mean of outside the field [−20, +20] μm was 12.57%. The deviations were not located in specific areas. However, it was observed in the central grooves of unprepared posterior teeth. The differences mean in GCS-true was 10.22±1.06 µ (P<0.001). While the deviations ratios mean of greater than +20 μm was 10.53%, most of them occurred in the buccal surface of the central incisor, and the buccal and distal surfaces of the molars, and on the left side more than the right, that is, the deviations increased by getting away from the center of the model. In addition, the mean of negative deviations greater than −20 µm was 10.31%, which occurred in the palatal surfaces of the same areas. The reason for these deviations in the GCS group may be attributed to the use of a standard impression tray, as there was no homogeneous distribution of the impression material, in addition to the absence of a transverse back wing of the tray connecting the two buccal wings.

The differences mean in CIS-prec was 14.05±2.01 µ (P < 0.002). Deviations of outside the field [−20, +20] µm were 32.09%. Moreover, these deviations were in the distal regions of the arch, especially the distal palatal surfaces of the last molars and the buccal surfaces of the anterior teeth in more than half of the sample. The differences mean in CIS-true was 16.15 ± 2.07 µ (P < 0.002). Where the deviations ratios mean of greater than +20µm was 18.69%, it occurred on the palatal surfaces of the anterior teeth and the buccal surfaces of the posterior teeth. In addition, the negative deviations ratios mean of greater than −20 µm was 19.95%, which did not occur in specific areas. A difference value of up to −72 μm occurred on the palatal surface of the second molar. This can be explained by the fact that scanning the positive model is simpler and more accurate than scanning the negative model due to the difficulty of reaching light into the deep areas and its reflection reaching the camera.

For DIS-prec, the differences mean was 38.22±15.23 µm (P < 0.006). Also, deviations ratios mean of outside the field [-20, +20] µm was 65.73%, did not occur in specific places, and were generalized over the entire dental arch. Deviations exceeded 180 µm in more than one area.

The differences mean in DIS-true was 35.19 ± 8.7 (P < 0.006). Also, the deviations ratios mean of greater than +20 µm was 35.31%. These deviations did not occur in specific regions. However, these deviations were at the buccal surfaces for most of the teeth and the distal buccal surfaces of the last molars in more than two-thirds of the sample. As well as, the negative deviations ratios mean of greater than −20 µm was 32.31%. These deviations did not occur in specific regions. However, it was noted that the highest deviations were found in the palatal surfaces of the teeth in more than two-thirds of the sample. Visual analysis showed lower deviations in trueness values in the anterior teeth, with the largest difference in premolars and molars compared to the reference model. The decrease in the trueness value in the DIS group compared to other groups may be the result of the algorithms of the intraoral scanner software. In addition, some researchers found that digital intraoral systems might be affected by the shape, geometry, and position of the teeth in the dental arch [14,23]. The reason for the presence of the highest differences in the distal areas of the arch may be attributed to the fact that there are no points beyond which it can be relied upon as reference points when scanning is conducted, as the oral scanner scans each part of the arch depending on the previously scanned area, which causes the deformation of the arch, i.e., the change of the distance between the two ends of the arch. While the extra-oral scanner captures all the visual points from one angle as a whole and then changes the shooting angle. Moreover, the intraoral scanner captures fewer points than the extra-oral scanner. The number of points resulting from scanning the entire dental arch with the reference scanner was more than 100 thousand points, while it was about 55 thousand points for the intraoral scanner.

Although the differences between all groups are statistically significant, they had no clinical significance as 0.050 mm was adopted as a criterion for clinical acceptance because the accuracy of transferring details for pouring materials is acceptable when the 0.050 mm width line is copied continuously and clearly [25].

When comparing the results of this study with the published articles on the accuracy of conventional and digital impressions, it was found that our study was consistent with a study conducted by János Vág et al. on the upper jaw of a full-tooth human cadaver which found that conventional impressions were more accurate than digital impressions. Despite the different intraoral scanning systems used [26]. Our results correspond to Ender's study, as it found that the deviation mean of the conventional impressions of (53 ± 2 μm) was significantly different from that of the Trios 3 (μ8 ± 156) and CS 3600 (29 ± 365) intraoral scanners; however, it was significantly lower than the values ​​for element 1 (531 ± 26), element 2 (246 ± 11), emerald (317 ± 13), Omnicam (174 ± 11), and Planscan (903 ± 49) [27]. In addition, this study agreed with the study of Malik et al., conducted on a silver-coated upper jaw model, the precision values for conventional impression, Trios, and Omnicam groups were (21.7 ± 5.4), (49.9 ± 18.3) and (36.5 ± 11.12) μm, respectively, and trueness values was (24.3 ± 5.7), (87.1 ± 7.9) and (80.3 ± 12.1) μm, respectively. Whereas, conventional impressions showed higher precision (P < 0.006) and trueness (P < 0.001) values compared to the digital impressions for both devices. There were no significant differences in precision (P = 0.153) or trueness (P = 0.757) between the two scanners. Visual analysis revealed local deviations along the palatal surfaces of the molars and incisal edges of the anterior teeth [28].

In contrast, our results differ from the study of Christine Keul and Jan-Frederik Güth, which showed the superiority of the DIS group over the CIS and GCS groups [29]. This may be due to the difference in the intra-oral scanner and the use of a metal rod in the reference cast.

The result of this study indicates that there is no clinical difference between the digital impression and the conventional impression scan and gypsum cast scan. However, the digital impressions show different patterns of deviation compared to other study groups.

## Conclusions

Within the limitations of this study, we found that the reference scanner provides accurate results that can be used as a standard reference for conventional and digital impression comparisons. As well as, the GCS group showed the highest accuracy, followed by the CIS group, and the DIS group, with a statistically significant difference between all groups. However, the differences between all study groups had no clinical significance.
